# Exploring Eating Disorder Topics on Twitter: Machine Learning Approach

**DOI:** 10.2196/18273

**Published:** 2020-10-30

**Authors:** Sicheng Zhou, Yunpeng Zhao, Jiang Bian, Ann F Haynos, Rui Zhang

**Affiliations:** 1 Institute for Health Informatics University of Minnesota Minneapolis, MN United States; 2 Department of Health Outcomes & Biomedical Informatics University of Florida Gainsville, FL United States; 3 Department of Psychiatry University of Minnesota Minneapolis, MN United States; 4 Department of Pharmaceutical Care & Health Systems University of Minnesota Minneapolis, MN United States

**Keywords:** eating disorders, topic modeling, text classification, social media, public health

## Abstract

**Background:**

Eating disorders (EDs) are a group of mental illnesses that have an adverse effect on both mental and physical health. As social media platforms (eg, Twitter) have become an important data source for public health research, some studies have qualitatively explored the ways in which EDs are discussed on these platforms. Initial results suggest that such research offers a promising method for further understanding this group of diseases. Nevertheless, an efficient computational method is needed to further identify and analyze tweets relevant to EDs on a larger scale.

**Objective:**

This study aims to develop and validate a machine learning–based classifier to identify tweets related to EDs and to explore factors (ie, topics) related to EDs using a topic modeling method.

**Methods:**

We collected potential ED-relevant tweets using keywords from previous studies and annotated these tweets into different groups (ie, ED relevant vs irrelevant and then promotional information vs laypeople discussion). Several supervised machine learning methods, such as convolutional neural network (CNN), long short-term memory (LSTM), support vector machine, and naïve Bayes, were developed and evaluated using annotated data. We used the classifier with the best performance to identify ED-relevant tweets and applied a topic modeling method—Correlation Explanation (CorEx)—to analyze the content of the identified tweets. To validate these machine learning results, we also collected a cohort of ED-relevant tweets on the basis of manually curated rules.

**Results:**

A total of 123,977 tweets were collected during the set period. We randomly annotated 2219 tweets for developing the machine learning classifiers. We developed a CNN-LSTM classifier to identify ED-relevant tweets published by laypeople in 2 steps: first relevant versus irrelevant (F_1_ score=0.89) and then promotional versus published by laypeople (F_1_ score=0.90). A total of 40,790 ED-relevant tweets were identified using the CNN-LSTM classifier. We also identified another set of tweets (ie, 17,632 ED-relevant and 83,557 ED-irrelevant tweets) posted by laypeople using manually specified rules. Using CorEx on all ED-relevant tweets, the topic model identified 162 topics. Overall, the coherence rate for topic modeling was 77.07% (1264/1640), indicating a high quality of the produced topics. The topics were further reviewed and analyzed by a domain expert.

**Conclusions:**

A developed CNN-LSTM classifier could improve the efficiency of identifying ED-relevant tweets compared with the traditional manual-based method. The CorEx topic model was applied on the tweets identified by the machine learning–based classifier and the traditional manual approach separately. Highly overlapping topics were observed between the 2 cohorts of tweets. The produced topics were further reviewed by a domain expert. Some of the topics identified by the potential ED tweets may provide new avenues for understanding this serious set of disorders.

## Introduction

### Background of Eating Disorders and Social Media

Eating disorders (EDs) are a prevalent type of mental illness affecting more than 30 million people across different age groups in the United States [1]. These disorders are commonly underdiagnosed and undertreated [2], and even among individuals who receive diagnosis and treatment, recovery takes a long time to achieve and remains elusive to many [3,4]. Unfortunately, there are serious consequences associated with EDs. Affected individuals often experience significant negative psychological, physical, and interpersonal effects of ED symptoms [5]. Although evidence-based interventions are available for EDs, they are not helpful for many, suggesting that they may not be targeting the correct psychological variables for these individuals [6]. Thus, it is important to gather additional information on the thoughts, emotions, and behaviors of individuals with EDs to identify treatment targets to improve or develop effective interventions for these populations [7].

During the past decade, the number of users of social media platforms, such as Twitter and Facebook, has increased sharply. These platforms provide the general public with opportunities to express their thoughts and opinions and share information about their daily lives, including their health information. This practice has yielded a large number of social media messages that may provide valuable information on a variety of health topics. The analysis of these messages could produce knowledge, permitting more sensitive and accurate education and intervention design in different areas of public health [8]. As such, applying data mining techniques to analyze Twitter data has become a popular methodological approach in health care research.

For instance, a study in 2011 used the Ailment Topic Aspect Model, incorporated with previous knowledge, to create structured disease information from tweets that was subsequently used for the surveillance of a series of different ailments [8]. In 2016, Xu et al [9] checked the frequency of discussions on cancer-related topics among Twitter users and found differences among different race and ethnicity groups. In 2019, Musaev et al [10] applied a latent Dirichlet allocation (LDA) topic model to tweets to examine public discussions about cardiovascular disease and found that state health departments play an important role in communicating with the public about cardiovascular health. We have also conducted a series of studies using Twitter data on various health-related topics, from detecting adverse events to studying laypeople’s discussion of human papillomavirus vaccination [11-17]. We have also conducted initial work on using Twitter data to identify discussion topics relevant to EDs. These studies indicate that the analysis of tweets on a particular health topic, coupled with information derived from user profiles, may facilitate novel knowledge discovery in these areas of medicine. EDs have become a popular topic on Twitter. Thus, data mining methods and tools that can more effectively and efficiently identify and analyze these tweets and associated user profiles may help advance the research on ED-related content on social media. In addition, these methods can be translated for use in research on social media use relevant to health-related topics.

### ED-Related Research and Gaps

Due to the self-protective nature of EDs, many individuals may not be willing to communicate about their experience of the disorder with others, potentially limiting the ability of researchers and clinicians to understand the factors that promote ED symptoms [18,19]. However, many individuals with an ED use social media to engage in a more open discourse about ED content with others with shared experiences [20]. Although data on Twitter are publicly available, few studies have explored public discussions about EDs. One study investigated how some Twitter accounts promote ED symptoms and the associated negative health consequences among Twitter users. They manually collected data from 45 ED-promotion Twitter accounts, including the profile information, the tweets posted by these accounts, and information about their followers. Through content analysis, they identified a list of ED-related keywords in these tweets and found a positive correlation between the percentages of ED-relevant tweets posted by the ED-promotion accounts and their followers [19]. Another study collected and reviewed ED-relevant tweets and manually classified the collected tweets into different subgroups to provide insights on EDs and to inform future web-based interventions for EDs [21]. These studies indicate that analysis of ED-relevant tweets may help to provide insight into factors that motivate ED behaviors, which may further be used to prevent and treat EDs. However, these studies mainly used keyword-searching strategies to collect ED-relevant tweets and analyzed the content of tweets through manual review. This approach is limited because it only permits analysis of a relatively small number of tweets within a limited time frame with compromised efficiency in content analysis. As a result, the obtained information may not be sufficiently comprehensive, and manual analyses may not be scalable. To improve these studies, computational methods are needed to identify and analyze ED-relevant tweets.

### Objective of the Study

To expand upon previous research on social media engagement among individuals with EDs, the focus of this study is to develop an automatic approach to better understand public perceptions and thoughts about EDs and ED-related behaviors using Twitter data. Specifically, a machine learning approach was developed to identify ED-relevant tweets, and a topic modeling method was implemented to analyze the content of the identified tweets. Potential ED-related factors, such as behaviors, thoughts, and mental status, were summarized through content analysis.

## Methods

### Overview of Experimental Pipeline

**Figure 1:**
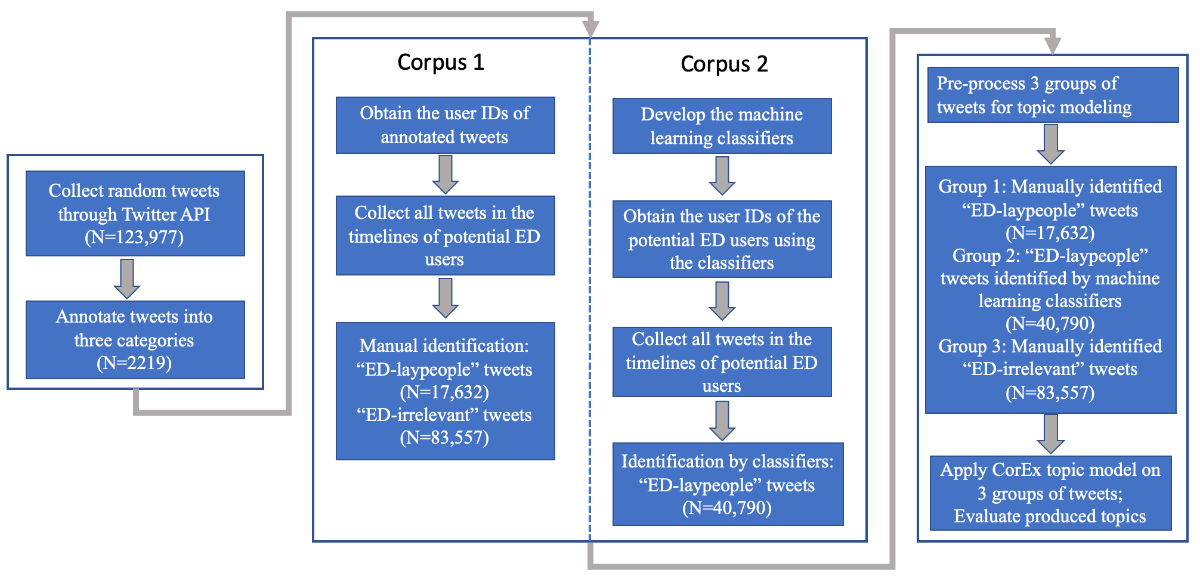
The workflow of the study. N represents the number of tweets at the corresponding step. CorEx: Correlation Explanation; ED: eating disorder.

The overall experimental pipeline is shown in Figure 1. We used 2 data sets spanning September 2012 to October 2019. We then randomly selected and annotated 2219 tweets to develop a training data set for the machine learning tasks of identifying ED-relevant tweets. We explored different machine learning–based methods to filter out ED-irrelevant tweets and classified the remaining tweets into either promotional and educational information or laypeople’s discussions. We determined if a tweet was published by a *layperson* on the basis of the content of the tweets. For example, if the tweet content was purely about an advertisement, the tweet was considered likely to be published by a *nonlayperson*. Thereafter, we performed topic modeling on 2 corpora. One corpus was built manually on the basis of 2219 annotated tweets and regarded as the gold standard for tweets that contain laypeople’s discussions about EDs. Another corpus of ED-relevant tweets was built using the developed machine learning classifiers. We evaluated the topic modeling results to validate whether the corpus built by our developed classifiers could produce similar ED-relevant topics compared with the manually identified gold standard corpus. We also further identified and analyzed topics from the ED-irrelevant tweets posted by the potential ED users.

### Twitter Data Collection

The Twitter data used in this study were from 2 different sources: (1) we used a list of ED keywords we developed from previous studies [19,21] (Textbox 1) and used these keywords to search for potential ED-relevant tweets from a database of random tweets collected from January 1, 2012, to September 30, 2018, using the Twitter streaming application programming interface (API); and (2) we used the same list of keywords to collect more Twitter data by using the Twitter search API from September 26, 2019, to October 30, 2019. This time span was selected because we have been collecting Twitter data since 2012.

**List of eating disorder–relevant keywords refined from previous studies.**

Eating disorder–relevant keywords:

- Anorexia
- Anorexic
- Anamia
- ana/mia
- Anahelp
- Anabuddy
- Anaprobs
- Binge
- Bulimia
- bulimic
- #BingeEatingDisorder
- #CompulsiveEating
- ednos
- edlogic
- edprobs
- edproblems
- pro ana
- proana
- pro mia
- promia
- purge

### Identification of Target Tweets

#### Annotation of Tweets

Of the 123,977 total tweets, we randomly selected 2219 tweets on the basis of keyword distribution for gold-standard data set development. The tweets were annotated into 3 categories: *ED-irrelevant*, *ED-promotional and education*, and *ED-laypeople*. *ED-irrelevant* tweets were tweets considered irrelevant to EDs, *ED-promotional and education* tweets were tweets considered to be published by companies and institutes to promote their products or educate the public about EDs, and *ED-laypeople* tweets were considered to be ED-relevant tweets posted by individual users. The tweets labeled with *ED-laypeople* were our target tweets, and the individual users who posted the *ED-laypeople* tweets were defined as potential ED users. Owing to the nature of social media, it is extremely difficult to determine which user does indeed have an ED. However, a large portion of these users were highly engaged in the discussion of ED symptoms or frequently posted their activities and thoughts about EDs and how ED symptoms affected their lives. Two annotators started with 100 tweets to develop an initial annotation guideline to identify the category of each tweet on the basis of its content. Thereafter, we annotated another 100 tweets to refine this guideline. Finally, each of the remaining 2019 tweets were annotated by 2 coders. Agreements were calculated, and conflicts were resolved through group discussions.

#### Manually Identified ED-Laypeople Tweets and ED-Irrelevant Tweets

Within the 2219 tweets, we extracted the Twitter user accounts that posted tweets in the *ED-laypeople* category. We manually reviewed the usernames and parts of their tweets and removed users whose usernames and tweets indicated that they were not *laypeople*; for example, if a username contained the name of a company or an institute, the tweets from that account would be classified as not belonging to the *ED-laypeople* category. Through this process, 31 accounts were removed for being companies or institutes. The remaining accounts were classified as potential ED users. We then collected all the tweets posted by these potential ED users to construct their Twitter timelines. We checked all of their tweets to see if these tweets contained one of the ED keywords in Textbox 1. If so, the tweets were regarded as *ED-laypeople* tweets, otherwise as *ED-irrelevant* tweets. The *ED-laypeople* tweets and the *ED-irrelevant* tweets were further analyzed using a topic modeling method. Although these *ED-irrelevant* tweets were classified as not directly associated with EDs, they were considered to reflect general topics within the potential ED users’ daily lives, which could help to differentiate ED-related experiences from other aspects of these users’ lives.

#### Machine Learning Classifier Development

Supervised text classifiers can learn patterns from annotated input samples and automatically classify tweets into desired categories. To develop the classifiers, we first preprocessed the annotated tweets by replacing (1) hyperlinks (eg, *http://t.co/xxxx*) with *<url>*, (2) mentions (eg, *@username*) with *<user>*, (3) hashtags (eg,*#eatdisorder*) with *<hashtag> eatdisorder*, and (4) emojis with *<emojies>*. Then we explored 2 supervised deep learning algorithms (ie, convolutional neural network [CNN] and long short-term memory [LSTM]) and 5 machine learning algorithms (ie, naïve Bayes [NB], linear regression, support vector machine [SVM], random forest [RF], and gradient boosting trees [GB]) to classify our large tweet corpus automatically. We developed our classifiers in a 2-step process using the annotated tweets so that each classifier produced a binary output. In the first task, classifiers were trained to distinguish between ED-relevant and ED-irrelevant tweets (*ED-irrelevant* vs the union of *ED-promotional and education* and *ED-laypeople*) to filter out irrelevant tweets. In the second task, classifiers were trained to distinguish between the *ED-promotional and education* and *ED-laypeople* tweets. We developed a CNN classifier for the first task. The architecture of the CNN model included an embedding layer, a convolutional layer, a global max pooling layer, and a sigmoid output dense layer. We initialized the embedding layer with the Global Vectors for Word Representation pretrained 200-dimension Twitter word embeddings. In the convolutional layer, we set the number of filters to 64, the length of filters to 3, and the dropout rate to 0.2. For the second task, we developed an LSTM model. The architecture of the LSTM model included an embedding layer, an LSTM layer, a global max pooling layer, and a sigmoid output dense layer.

### Identifying Topics From Tweets of Potential ED Users

To understand the mental status and everyday life of potential ED users, we applied topic modeling to explore their tweets. Topic modeling has been a popular method for identifying latent patterns of words in a large collection of documents [22]. The most representative method for topic modeling is LDA—a probabilistic generative model [23]. In LDA, each document is assumed to contain a mixture of topics, where each topic is a probability distribution over the words in the document [24]. Some new topic models were developed to solve some of the limitations in LDA, such as the Biterm Topic Model (BTM) and the Correlation Explanation (CorEx) model [24,25]. BTM mainly improves the LDA’s problem of sparse word co-occurrence patterns at the document level; thus, it uses the term *co-occurrence patterns* in the entire corpus to learn topics [24]. The CorEx model does not have an assumption about how the underlying data are generated, similar to LDA, which avoids assigning the characteristics of topics ahead of time. The CorEx model identifies the topics that are *maximally informative* about a collection of documents [25]. The BTM and the CorEx model were tested in our preliminary study [17], and the CorEx model was adopted because it produced more meaningful topics using our collected tweets.

We implemented the CorEx model on 3 groups of Twitter data: (1) the *ED-laypeople* tweets identified through manually curated rules posted by potential ED users, (2) the *ED-irrelevant* posted by potential ED users, and (3) *ED-laypeople* tweets identified by the machine learning algorithm, as mentioned earlier. For each group, we tested the CorEx model with different numbers of topics (n=50, 60, 70, 80, 90, 100). Although quantitative metrics are used to infer a reasonable number of topics (eg, perplexity and coherence), they sometimes cannot identify the optimal number of topics. On the basis of our experience in a previous study [17], we manually reviewed the topics produced by the different experiments to determine the optimal number of topics for further topic evaluation.

### Topic Evaluation

The results of the topic modeling experiments were further reviewed and analyzed by the domain expert (AH). Three steps were taken to evaluate the topic modeling results. First, the domain expert summarized the theme for each topic on the basis of the topic keywords. Second, on the basis of the top 10 most-relevant tweets for each topic, the expert judged whether each tweet was coherent with the summarized topic theme. The coherence rate of each topic, defined as the percentage of coherent tweets per topic, was calculated. Finally, topic themes with similar meanings were merged into higher-level categories.

## Results

### Tweet Collection and Annotation

Two coders annotated 2219 tweets into 3 classes, as mentioned earlier. Within 2219 tweets, 669 tweets were annotated as *ED-irrelevant*, 579 tweets were annotated as *ED-promotional and education*, and 971 tweets were annotated as *ED-laypeople*. The interrater agreement score between the 2 annotators was 0.84 on the basis of the first 200 tweets. We used the Cohen kappa test to calculate the score.

### Identification of the Target Tweets

#### Manual Identification of ED-Laypeople and ED-Irrelevant Tweets

As described earlier, we manually identified 17,632 *ED-laypeople* tweets and 89,312 *ED-irrelevant* tweets posted by the potential ED users.

#### Machine Learning Classifier Development

As mentioned earlier, 7 classifiers were explored (ie, CNN, LSTM, NB, LN, SVM, RF, and GB). We developed our classifiers in a 2-step process (ie, *ED-irrelevant* vs the other 2 labels and then *ED-promotional and education* vs *ED-laypeople*). Overall, 79.99% (1775/2219) of the tweets were used as the training set, and 20.01% (444/2219) tweets were used for evaluation. Table 1 shows the performances of the classifiers. 

**Table 1:** Performances of the developed classifiers.

Classifier		Precision	Recall	F_1_ score	*P* value
***ED*^a^*-irrelevant* versus other 2 labels^b^**					
	*CNN^c^*	*0.88*	*0.89*	*0.89*	N/A^d^
	LSTM^e^	0.86	0.89	0.88	.15
	NB^f^	0.85	0.73	0.75	<.001
	LN^g^	0.84	0.78	0.81	<.001
	SVM^h^	0.87	0.83	0.85	<.001
	RF^i^	0.86	0.85	0.86	.005
	GB^j^	0.77	0.75	0.76	<.001
***ED-promotional and education* versus *ED-laypeople*^k^**					
	*LSTM*	*0.90*	*0.89*	*0.90*	N/A
	CNN	0.87	0.87	0.87	.006
	NB	0.80	0.74	0.76	<.001
	LN	0.83	0.80	0.81	<.001
	SVM	0.82	0.79	0.80	<.001
	RF	0.84	0.82	0.83	<.001
	GB	0.84	0.82	0.83	<.001

^a^ED: eating disorder.

^b^ED-irrelevant versus other 2 labels: in this task, the performances of CNN and LSTM have no significant difference; they are both significantly higher than the others (*P*<.01).

^c^CNN: convolutional neural network.

^d^N/A: not applicable.

^e^LSTM: long short-term memory.

^f^NB: naïve Bayes.

^g^LN: linear regression.

^h^SVM: support vector machine.

^i^RF: random forest.

^j^GB: gradient boosting trees.

^k^ED-promotional and education versus ED-laypeople: in this task, the performance LSTM is significantly higher than the others (*P*<.01).

In the first task, the CNN outperformed the other classifiers (F_1_ score=0.89). The CNN classifier identified 88,261 tweets that were ED-relevant. In the second task, LSTM obtained the best performance (F_1_ score=0.90). Thus, we adopted LSTM for the second task. The LSTM method identified 40,790 *ED-laypeople* tweets posted by 21,600 Twitter users.

### CorEx Topic Model Implementation

The CorEx topic model was implemented on 3 groups of Twitter data, as mentioned earlier. After preprocessing, the first group (*ED-laypeople* tweets manually identified) contained 17,632 tweets. The second group (*ED-laypeople* tweets identified by the developed CNN-LSTM classifier) contained 40,790 tweets. The third group (*ED-irrelevant* tweets posted by the potential ED users) contained 83,557 tweets. After the initial review, the optimal number of topics was determined to be 70 for groups 1 and 2. For group 3, the optimal number of topics was determined to be 80. Textbox 2 shows the representative words of selected topics obtained from 3 groups of tweets.

**Representative words of selected topics obtained from 3 groups of tweets.**

Weight loss

- Weight, lose, lost, gain, lb

Eating disorder symptoms

- Purge, binge, crave, buffet, bathroom

Food and drink

- Coke, breakfast, sandwich, chicken, yogurt

Body image

- Collar bone, thigh, fat, mirror

Media or advertising or portrayals

- Instagram, twitter, media, social, tumblr

Mental illness

- Mental, ill, breakdown, think, disorder

Negative consequences

- Sleep, hunger, stress, escape, pain

Negative emotions

- Depress, apart, alone, sad, pointless

Education or awareness or treatment

- Therapist, session, save, visit, came

Recovery

- Battle, strength, courage, stronger, inspiring

### Topics Evaluation

The CorEx model results of the 3 experimental groups were reviewed and analyzed by a domain expert (AH). For group 1, which used manually identified *ED-laypeople* tweets, 54 of 70 topics were identified as meaningful, and each of them was assigned a topic theme. Similar themes were further grouped into 15 higher-level categories. The top 10 relevant tweets of each topic were reviewed and judged whether they were coherent with the summarized topic theme, and the coherence rate was calculated. Table 2 lists the summary of group 1, including the identified higher-level topic categories, the number of topic themes under each category, some representative topic themes, and the coherence rates for each topic category.

For the second group that used *ED-laypeople* tweets identified by the developed classifier, 63 of 70 topics were identified as meaningful topics and were assigned topic themes. The 63 topics were further merged into 19 categories. Table 3 shows a summary of the topics in the second group, including the identified topic categories, the number of topic themes under each category, example of representative topic themes, and the coherence rates for each category.

For the third group, which used manually identified *ED-irrelevant* tweets posted by the potential ED users, 47 of 80 topics were reviewed as significant topics. The 47 topics were further merged into 19 categories. Table 4 shows a summary of the results from group 3.

Compared with our previous study [17], several new topics were identified, including *Questions or Concerns*, *Reflection or Planning*, *Comorbidity*, *Ambivalence*, *Insults*, and *Diagnostic criteria.* Textbox 3 shows these topic themes and example tweets.

We also explored the ED-irrelevant tweets posted by the potential ED users (ie, group 3). Selected topics and relevant tweets are listed in Textbox 4.

**Table 2:** A summary of the topics using group 1 tweets (ie, manually identified ED-laypeople tweets).

Topic category	Population, n	Number of topics under each category	Representative topic themes	Coherence rate, n (%)
ED^a^ recovery	90	9	Learning from the past; Hope; Moving forward	69 (77)
ED symptoms	70	7	Weight loss and gain; Binge-eating and purging	61 (87)
Education or awareness or treatment	60	6	ED education; ED treatment	51 (85)
Random words	60	6	Love; Big; Life; Rock	48 (80)
Negative consequences	50	5	Health damage; Feeling trapped	36 (72)
Body image	50	5	Collar bones; Thinness	39 (78)
Food and drink	30	3	Food and drink	23 (77)
Pro-ana	30	3	Pro-ana	24 (80)
Negative emotions	30	3	Guilt and shame; Fear; Sadness	23 (77)
Media or advertising or portrayals	20	2	Media and advertising	12 (60)
Comorbidity	10	1	Comorbidity	10 (100)
Reflection or planning	10	1	Reflection or planning	9 (90)
Ambivalence	10	1	Ambivalence	8 (80)
Diagnostic criteria	10	1	Diagnostic criteria	7 (70)
Questions or concerns	10	1	Questions or concerns	10 (100)

^a^ED: eating disorder.

**Table 3:** A summary of the topics using group 2 tweets (ie, ED-laypeople tweets identified by the developed classifiers).

Topic category	Population, n	Number of topics under each category	Representative topic themes	Coherence rate, n (%)
ED^a^ symptoms	100	10	Restriction; Appetite suppression; Binge-eating; Purging	65 (65.0)
Education or awareness or treatment	90	9	ED education; Support group	76 (84.4)
Media or advertising or portrayals	90	9	Media or advertising	65 (72.2)
ED recovery	80	8	Passion; Hope; Love	63 (78.8)
Negative consequences	70	7	Health damage; Social	44 (62.9)
Food and drink	20	2	Food and drink	13 (65.0)
Social media	20	2	Twitter; Social media	11 (55.0)
Pro-ana	20	2	Pro-ana	16 (80.0)
Insults	20	2	Insults	18 (90.0)
Reflection or planning	20	2	Reflection or planning	17 (85.0)
Comorbidity	20	2	Comorbidity	19 (95.0)
Mental illness	10	1	Mental illness	9 (90.0)
Negative emotions	10	1	Negative emotions	8 (80.0)
Body image	10	1	Body image	8 (80.0)
Weight extremes	10	1	Weight extremes	5 (50.0)
Negative social reactions	10	1	Negative social reactions	8 (80.0)
Diagnosis	10	1	Diagnosis	9 (90.0)
Questions or concerns	10	1	Questions or concerns	6 (60.0)
Random words	10	1	Anger	9 (90.0)

^a^ED: eating disorder.

**Table 4:** A summary of the topics using group 3 tweets (ie, manually identified ED-irrelevant tweets posted by the potential ED users).

Topic category	Population, n	Number of topics under each category	Representative topic themes	Coherence rate, n (%)
Negative emotions or attitude	80	8	Negative emotion; Pressure; Hate	68 (85)
ED^a^ behaviors	70	7	Restriction; Purging; Laxatives	55 (79)
Body image	50	5	Hair; Appearance	36 (72)
Exercise	40	4	Exercise	26 (65)
Media	30	3	Entertainment; Music	20 (67)
Self-harm	20	2	Self-harm	17 (85)
Negative consequences	20	2	Mental health damage	19 (95)
Communication	20	2	Seeking communication; Connection	12 (60)
Weight loss	20	2	Weight loss; Concern about weight	18 (90)
Positive emotions	20	2	Positive emotions; Encouraging	14 (70)
Holidays	20	2	Christmas; Halloween	15 (75)
Food and drink	10	1	Food and drink	9 (90)
Social media	10	1	Social media	8 (80)
Suicide	10	1	Desire for suicide	9 (90)
Appreciation	10	1	Appreciation	10 (100)
Sleep deprivation	10	1	Sleep deprivation	10 (100)
Grocery or shopping	10	1	Grocery or shopping	6 (60)
Intimate relationships	10	1	Intimate relationships	7 (70)
School	10	1	Negative social reactions	6 (60)

^a^ED: eating disorder.

**New topics identified from ED-laypeople tweets in groups 1 and 2.**

Comorbidity

- “#mentalhealthawareness We're hiring. I myself suffer with 6 mental health illnesses. Anxiety, Depression, OCD, BPD, Anorexia and PTSD”
- “Got my full diagnosis list. I have major depression with psychotic features, GAD, anorexia nervosa, binge eating, purging type, ptsd, and bpd”
- “Literally my everyday life, so many diagnosis, ocd, did, bulimia, anorexia ....anxiety depression until 11 years later “bpd” or eupd as they call it in uk, its hard to tell ppl more should be done to raise awareness so much praise for @xxxxxxx”

Reflection or planning

- “A few months ago I was hiding in my dorm room severely depressed and relapsing from anorexia; tomorrow morning marks one month of me taking recovery seriously; is also my move-in day at a new school & I am so excited to get healthy again and for a new start and I am truly happy”
- “So yesterday was a struggle with Candy it was all around constantly and I caved . :( but I will get back on track today. I will pretend to be sick on thanksgiving and on Christmas Eve that way I don’t get tempted by candy .... I’m done with looking at that scale go up. #proana”
- “Live for today, let yesterday go, and keep smiling for tomorrow. #Anorexia #ED #life #strength #dontgiveup”

Insults

- “Net of 0 today. My mom made me eat a lean cuisine :( she’s yelling all day that I’m “anorexic”. Stfu, leave me alone I’m FAT.”

Diagnostic criteria

- “@xxxxxx even at my sickest, I didn’t meet the weight criteria for anorexia diagnosis. But I had severe muscle wastage at that point”
- “@xxxxxxx what a sweetheart you are. I was quoting the medically accepted diagnostic criteria for anorexia nervosa... thx for the information.”

Ambivalence

- “@xxxxxx @xxxxx Hi I also have an eating disorder, and I often find it comforting. Please don’t speak on behalf of every single person with an ED. We are all very different, and you never know what helps. Let’s try building each other up.”
- “Worst feeling ever is missing my thin anorexia body even though logically I know it was unhealthy and killing me. And now I have to struggle everyday to live in this “fat” body. Just completely unbearable at times.”
- “My eating disorder is my worst enemy, yet my closest friend.”

**The selected topics and the representative tweets in group 3.**

Negative emotions or attitudes

- “There was a certain weight threshold I never wanted to go above. And now I’m above that. I feel like a complete utter failure.”
- “Honestly I’m disgusting. How can I even contemplate eating when I’m this morbidly obese. Jesus.”
- “Sorry for being bitchy guys. I’m really tired, I need a shower, I’m worried, and I’m hungry. I’m just grumpy.”

Eating disorder symptoms

- “Haven’t eaten in 25hours, will eat in 30minutes. Salad & Chicken.”
- “I haven’t properly fasted for such a long time, only restricted or binged. My fast began 3 hours ago and I’m actually excited! #fasting”
- “@xxxxx I’m liquid fasting for at least 24hrs now.”

Self-harm

- “My wrist n cuts are getting dry, they disappear so fast”
- “I have so many cuts at the moment. I went a bit mental last week. Everything’s just falling apart.”
- “All because I’m so emotional, I'm gonna be flaunting some major self harming. The county fair is next week... Everyone will see...”

Negative consequences

- “I feel so empty, I just want to cut my wrist and bleed out #depressed #depressedgirl #nomoreburns #numb”
- “crying. life and mind are falling apart again.”
- “The self hatred is so mentally and physically draining.”
- “I feel so mentally unstable at the moment. Like I’m on the verge of a complete mental breakdown any minute.”

Communication

- “Someone talk to me please. I’m just going mad please!”
- “Someone please talk me out of the Popsicle I want so bad. #HelpMe”
- “I could use someone to talk to. By talk, I mean text lol feelin kinda lonely all alone. :( #miasisters”

Suicide

- “I'm getting recurrent suicidal thoughts. My mental health is just awful at the moment. I feel very self destructive and unstable. I hate it.”
- “I want to die so bad but having to commit suicide will change my family’s life and other peoples outlook on them”
- “I’m so depressed, I feel like the only escape is suicide. Life is so pointless, you live in mental hell, you live with physical pain, you end up alone hen death.”

Sleep deprivation

- “It’s 4:40am and I can’t fall asleep. I'm yawning cause I’m tired, but I can’t find sleep. I have too many things going through my mind.”
- “Laying in bed and my stomach won’t stop churning, think I’m gonna be having a restless night tonight!”
- “In the past two days I’ve drank 23 cups of coffee and actually hallucinated. I’m not sure if its from sleep deprivation or caffeine #Whoops”

Laxative usage

- “Uh-oh! Can start to feel the lax cramps now......”
- “I can start to feel my laxatives kicking in. I took 5 so far. Just hate the cramps when I take laxatives. :-/”
- “I’ve been using a lot of laxatives lately. It doesn’t seem to do much except give me cramps.”

## Discussion

### Identification of ED-Relevant Tweets and Topic Modeling

In earlier studies, the identification of ED-relevant tweets was based on a manual search method using ED keywords or hashtags and filtered by manually curated rules [19,21]. This approach was not efficient and may have included numerous irrelevant tweets owing to the ambiguity of the keywords. In this study, we developed deep learning classifiers to automatically identify relevant tweets posted by *ED-laypeople*. We simplified the classification task into a 2-step process and achieved good performance with 2 supervised deep learning classifiers (ie, CNN for step 1 and LSTM for step 2) and obtained reasonable results with F_1_ scores of 0.89 and 0.90, respectively. For content analysis of the identified tweets, we applied topic modeling, an unsupervised method, and manual review, which could summarize comprehensive information from tens of thousands of tweets. This approach is more efficient compared with the completely manual review, which could only cover several hundreds of tweets [21].

### Analysis of Topic Modeling Results

The overall coherence rate for the 3 experimental groups was 77.07% (1264/1640), which indicates high quality of the topics produced by the CorEx model. The categories and higher-level themes of the produced topics were summarized by the domain expert (AH). Some of these categories may seem unclear or disparate if not considered within the right context. For example, the *Insults* category referred to using ED symptoms or terms to insult someone (eg, using the word *anorexic* as a derogatory term). Therefore, this content was ED-related, although not pertaining to a traditional ED topic (eg, ED symptoms). This principle was similar for the other categories. For instance, the category of *Questions* referred to a range of ED content (eg, “@instagram This is not a difficult question - how do I escalate a complaint about dangerous, self-harm-encouraging, pro ana content that your algorithm has deemed safe?”), although the organizing principle for this group of Tweets was question-asking (ie, all tweets were posed in the form of a question). We believe that the diversity of topics identified by this algorithm is a strength of this investigation. Producing a range of topics will allow researchers to better understand the social media content on EDs, including content that would not have been expected. This information could ultimately inform future mechanistic and treatment research on EDs. For instance, we would not have anticipated that ED terminology would be used as insults on social media. We hypothesize that this type of language could further stigmatize this set of disorders, which present a target for future prevention efforts. In addition, the *Questions* category identified issues important to potential ED users on social media, such as the need for better social media monitoring and blocking of content that could be detrimental to individuals with, or vulnerable to developing, an ED. This further highlights the importance of generating algorithms for identifying ED-related content on social media. Improvement of these methods could be used by social media platforms such as Twitter to improve filtering practices for harmful content and/or to provide appropriate mental health resources to individuals posting or viewing such content.

The first and second groups focused on the *ED-laypeople* tweets posted by manually identified potential ED users. One aim of comparing these 2 groups was to verify that the *ED-laypeople* tweets identified by manually specified rules and a machine learning classifier could produce similar topics. The 2 groups were found to have 14 overlapping topic categories, such as *Negative consequences*, *ED symptoms*, *Education or awareness or treatment*, and *ED recovery*. This result was in agreement with our previous study [17]. There were slight discrepancies in the identified categories between the 2 groups. *Body image* uniquely appeared among the highest frequency topics in group 1, whereas *Media or advertising or portrayals* uniquely appeared among the highest frequency topics in group 2. The higher prevalence of the *Media or advertising or portrayals* topic category in group 2 reveals a pitfall of the developed machine learning classifier; some *ED-promotional and education* tweets were misclassified as *ED-laypeople* tweets by the classifier, leading to a larger *Media or advertising or portrayals* topic category. When we manually identified the *ED-laypeople* tweets, we could check both the content of the tweets and the user profile of the accounts that posted the tweets. However, the developed classifier only used the content from the tweets themselves and did not incorporate profile information. Including user information such as usernames as features in the classifier would have the potential to mitigate this misclassification problem. Furthermore, group 1 had one unique topic category, *Ambivalence*, occupying 2% (1/54) of total meaningful topics in this group, whereas group 2 had unique categories of *Negative social reactions,* *Insults,* *Mental illness,* and *Social media,* occupying 10% (6/63) of the total meaningful topics. In general, the 2 groups of tweets produced highly similar topics, indicating that our machine learning classifier was mostly as effective as the manual method. This is meaningful because it suggests that this method could be used to identify ED-relevant social media content for future larger scale investigations. In addition, such methods could ultimately aid in identifying at-risk groups for whom prevention efforts could be targeted, which is especially important given the low level of ED detection in typical practice [2].

According to Textbox 4, 6 new topics were identified compared with our previous study [17]. These topics are consistent with earlier literature on EDs and may provide novel insights into the thoughts and experiences of individuals with EDs. Several areas of the content reflected topics that may be relevant to understanding the decisional mechanisms involved in these disorders. The *Reflection or planning* category (eg, “A few months ago I was hiding in my dorm room severely depressed and relapsing from anorexia; tomorrow morning marks one month of me taking recovery seriously; is also my move-in day at a new school.”) and *Ambivalence* category (eg, “My eating disorder is my worst enemy, yet my closest friend.”) reflect the strong pull of individuals with EDs toward and against ED symptoms [26]. This suggests that for some, the decision about whether to engage in ED behavior involves consideration of the pros and cons of engaging in these behaviors and planning future actions [26]. In future research, it would be informative to determine whether individuals producing tweets reflecting the deliberative processes of weighing pros and cons of ED behaviors vary in a clinically relevant manner from those not producing these tweets, as there is a suggestion that deliberative processes might characterize earlier stages of illness [18]. In addition, the *Comorbidity* and *Diagnostic criteria* categories highlight the importance of considering heterogeneity across a range of severity and symptom profiles in ED research and treatment [27]. Many ED researchers have highlighted the importance of considering certain ED characteristics (eg, whether a person is emotionally dysregulated vs constrained) in planning treatments [28]. In addition, it has long been acknowledged that *Diagnostic criteria* categories fail to capture many individuals who do not present with classic ED symptoms (eg, meet all criteria for anorexia nervosa but are not underweight) [29]. These topics also might characterize content from clinically unique subgroups of individuals with EDs, warranting further consideration.

The summarized topics for the group 3 tweets are listed in Textbox 3. The high-frequency topics (n>5) identified from the ED-irrelevant tweets included *Negative emotions or attitudes* (n=8), *Eating disorder behaviors* (n=7), and *Body image* (n=5). The content identified in these analyses reveals what other topics are common to potential ED users in their daily lives. One notable feature of these results is that the content of many of the tweets identified as *ED-irrelevant* pertains explicitly to ED cognitive and behavior symptoms (eg, *ED symptoms* and *Body image* categories). One interpretation is that for individuals with ED, the experience with their disorder becomes so pervasive that it infiltrates content that is not explicitly intended as ED-relevant. However, this could also indicate that the algorithm needs to be further refined to capture all the content relevant to EDs. An additional finding from this analysis is that many of the tweets demonstrate that negative emotion and pain predominate the experience of having an ED, as reflected in the categories *Negative emotions or attitude*, *Negative consequences*, *Self-harm*, and *Suicide*. These categories correspond with theories that ED symptoms may result from a surfeit of negative emotions and that the symptoms themselves may function to alleviate emotional pain in a similar fashion to self-harm and suicide planning [30-32].

### Limitations

We developed machine learning classifiers that could identify ED-relevant tweets with high performance, but there is still a small percentage of misclassified tweets, especially in the task of differentiating the *ED-promotional and education* versus *ED-laypeople* tweets. This may be partially due to the short length of some tweets, which makes them difficult to classify. Furthermore, these 2 types of tweets are sometimes semantically similar. With the increase in the number of collected tweets, there will be a large number of misclassified tweets, although the misclassification rate is low, which will further influence the results of topic modeling as it will produce some noise topics. Another limitation is that we cannot collect all the tweets in the entire timeline of our target potential ED users owing to the restriction of Twitter API, which may lead to other useful topics being missed.

### Conclusions

Our study developed a 2-step process using 2 classifiers (ie, CNN and LSTM) that could automatically identify ED-relevant tweets posted by the potential ED users. The F_1_ scores of the 2 classifiers were 0.89 and 0.90, respectively. A CorEx model was applied on the tweets identified by the classifiers and those identified by a traditional manual method separately. Highly overlapping topics were produced. Through a review of these topics by a domain expert, important features of the social media content of potential ED were identified. These findings provided novel insights into the experience of having an ED, which could be expanded upon in future research using the methods derived in this investigation.

## Abbreviations

API: application programming interface
BTM: Biterm Topic Model
CNN: convolutional neural network
CorEx: Correlation Explanation
ED: eating disorder
GB: gradient boosting trees
LDA: latent Dirichlet allocation
LSTM: long short-term memory
NB: naïve Bayes
NIH: National Institutes of Health
NSF: National Science Foundation
PI: principal investigator
RF: random forest
SVM: support vector machine

## References

[1] Hudson JI, Hiripi E, Pope HG, Kessler RC (2007). The prevalence and correlates of eating disorders in the National Comorbidity Survey Replication. Biol Psychiatry.

[2] Kutz AM, Marsh AG, Gunderson CG, Maguen S, Masheb RM (2020). Eating Disorder Screening: a Systematic Review and Meta-analysis of Diagnostic Test Characteristics of the SCOFF. J Gen Intern Med.

[3] Eddy KT, Tabri N, Thomas JJ, Murray HB, Keshaviah A, Hastings E, Edkins K, Krishna M, Herzog DB, Keel PK, Franko DL (2017). Recovery From Anorexia Nervosa and Bulimia Nervosa at 22-Year Follow-Up. J Clin Psychiatry.

[4] Steinhausen H (2002). The outcome of anorexia nervosa in the 20th century. Am J Psychiatry.

[5] Bauer S, Kindermann SS, Moessner M (2017). [Prevention of eating disorder: a review]. Z Kinder Jugendpsychiatr Psychother.

[6] Berkman ND, Bulik CM, Brownley KA, Lohr KN, Sedway JA, Rooks A, Gartlehner G (2006). Management of eating disorders. Evid Rep Technol Assess (Full Rep).

[7] Kass AE, Kolko RP, Wilfley DE (2013). Psychological treatments for eating disorders. Curr Opin Psychiatry.

[8] Paul MJ, Dredze M (2011). You are what you tweet: Analyzing Twitter for public health.

[9] Xu S, Markson C, Costello KL, Xing CY, Demissie K, Llanos AA (2016). Leveraging Social Media to Promote Public Health Knowledge: Example of Cancer Awareness via Twitter. JMIR Public Health Surveill.

[10] Musaev A, Britt RK, Hayes J, Britt BC, Maddox J, Sheinidashtegol P (2019). Study of Twitter communications on cardiovascular disease by state health departments.

[11] Bian J, Topaloglu U, Yu F (2012). Towards large-scale twitter mining for drug-related adverse events.

[12] Zhang H, Wheldon C, Dunn AG, Tao C, Huo J, Zhang R, Prosperi M, Guo Y, Bian J (2020). Mining Twitter to assess the determinants of health behavior toward human papillomavirus vaccination in the United States. J Am Med Inform Assoc.

[13] Modave F, Zhao Y, Krieger J, He Z, Guo Y, Huo J, Prosperi M, Bian J (2019). Understanding Perceptions and Attitudes in Breast Cancer Discussions on Twitter. Stud Health Technol Inform.

[14] Zhao Y, Guo Y, He X, Wu Y, Yang Xi, Prosperi M, Jin Y, Bian J (2020). Assessing mental health signals among sexual and gender minorities using Twitter data. Health Informatics J.

[15] Wang Y, Zhao Y, Zhang J, Bian J, Zhang R (2020). Detecting associations between dietary supplement intake and sentiments within mental disorder tweets. Health Informatics J.

[16] Hicks A, Hogan WR, Rutherford M, Malin B, Xie M, Fellbaum C, Yin Z, Fabbri D, Hanna J, Bian J (2015). Mining Twitter as a First Step toward Assessing the Adequacy of Gender Identification Terms on Intake Forms. AMIA Annu Symp Proc.

[17] Zhou S, Bian J, Zhao Y, Haynos AF, Rizvi R, Zhang R (2019). Analysis of Twitter to Identify Topics Related to Eating Disorder Symptoms. IEEE Int Conf Healthc Inform.

[18] Walsh BT (2013). The enigmatic persistence of anorexia nervosa. Am J Psychiatry.

[19] Arseniev-Koehler A, Lee H, McCormick T, Moreno MA (2016). #Proana: Pro-Eating Disorder Socialization on Twitter. J Adolesc Health.

[20] Kenny TE, Boyle SL, Lewis SP (2020). #recovery: Understanding recovery from the lens of recovery-focused blogs posted by individuals with lived experience. Int J Eat Disord.

[21] Cavazos-Rehg PA, Krauss MJ, Costello SJ, Kaiser N, Cahn ES, Fitzsimmons-Craft EE, Wilfley DE (2019). "I just want to be skinny": A content analysis of tweets expressing eating disorder symptoms. PLoS One.

[22] Alghamdi R, Alfalqi K (2015). A Survey of Topic Modeling in Text Mining. Int. J. Adv. Comput. Sci. Appl.

[23] Barde BV, Bainwad AM (2017). An overview of topic modeling methods and tools.

[24] Yan X, Guo J, Lan Y, Cheng X (2013). A biterm topic model for short texts.

[25] Gallagher RJ, Reing K, Kale D, Ver Steeg G (2017). Anchored Correlation Explanation: Topic Modeling with Minimal Domain Knowledge. Transactions of the Association for Computational Linguistics.

[26] Serpell L, Treasure J, Teasdale J, Sullivan V (1999). Anorexia nervosa: friend or foe?. Int J Eat Disord.

[27] Wildes JE, Marcus MD (2013). Alternative methods of classifying eating disorders: models incorporating comorbid psychopathology and associated features. Clin Psychol Rev.

[28] Wildes JE, Marcus MD, Crosby RD, Ringham RM, Dapelo MM, Gaskill JA, Forbush KT (2011). The clinical utility of personality subtypes in patients with anorexia nervosa. J Consult Clin Psychol.

[29] Dunn EC, Geller J, Brown KE, Bates ME (2010). Addressing the EDNOS issue and improving upon the utility of DSM-IV: classifying eating disorders using symptom profiles. Eur Eat Disord Rev.

[30] Haynos AF, Fruzzetti AE (2011). Anorexia nervosa as a disorder of emotion dysregulation: evidence and treatment implications. Clin Psychol Sci Prac.

[31] Pisetsky EM, Haynos AF, Lavender JM, Crow SJ, Peterson CB (2017). Associations between emotion regulation difficulties, eating disorder symptoms, non-suicidal self-injury, and suicide attempts in a heterogeneous eating disorder sample. Compr Psychiatry.

[32] Wang SB, Borders A (2018). The unique effects of angry and depressive rumination on eating-disorder psychopathology and the mediating role of impulsivity. Eat Behav.

